# Assessing the Effects of Soil and Water Conservation Practices on Soil Erosion and Soil Fertility in the Fetam Watershed in the Blue Nile Basin of Ethiopia

**DOI:** 10.1155/tswj/8820426

**Published:** 2026-05-12

**Authors:** Sewyalew Tadele, Eyasu Elias, Abreham Berta Aneseyee

**Affiliations:** ^1^ College of Computational and Natural Sciences, Center of Environmental Science, Addis Ababa University, Addis Ababa, Ethiopia; ^2^ College of Agriculture and Natural Resource, Department of Natural Resource Management, Wolkite University, Wolkite, Ethiopia, wku.edu.et

**Keywords:** slope position, soil and water conservation practices, soil fertility, subwatersheds

## Abstract

Understanding sustainable land management is important for agricultural production. However, soil fertility has been declining due to anthropogenic and natural factors. To curb these problems, soil and water conservation (SWC) practices have been implemented over the last 15 years in the study watershed, but their impact has not been studied in detail. Therefore, this study was conducted to study the effects of SWC practices on soil fertility and soil erosion in the Fetam watershed. The Integrated Valuation of Ecosystem Services and Tradeoffs (InVEST) and sediment delivery ratio (SDR) model were used to analyze the sedimentation based on the required input parameters of the model such as land‐use/land‐coverraster data, Revised Universal Soil Loss Equation factors such as rainfall‐erosivity data, erodobilityfactor, cover factor (C), and management factors (P) and raster digital elevation model including the boundary of the study area in vector form. A total of 32 soil samples (0–20 cm) were collected and disaggregated by landscape position: upper, middle, and downstream. After collecting soil samples, the soil physicochemical properties, such as texture and bulk density (BD), and chemical properties such as (pH), organic carbon (OC), total nitrogen (TN), available phosphorus (AP), exchangeable bases, and cation exchnage capacity (CEC) were analyzed using standard laboratory procedures in the treated (Hunkan) and untreated (Kindikan) subwatersheds. The *t*‐test and analysis of variance (ANOVA) results show that the physical and chemical properties of soil differed significantly between treated and untreated subwatersheds and between upper and lower landscape positions (*p* < 0.05). The mean soil loss in treated and untreated microwatersheds was 14.43 ± 6.63 and 20.31 ± 10.28 t/ha, respectively. This indicates that the SWC structures implemented in the treated subwatershed contributed to reducing soil erosion rates. The treated watershed and lower landscape position had the highest OC (3.69 ± 0.23) and the lowest BD (1.05 ± 0.01). In contrast, the untreated watershed and upper landscape position had the highest BD (1.21 ± 0.01) and the lowest OC (2.35 ± 0.23). Overall, the soil fertility parameters were most favorable in the treated fields and lower‐slope positions, suggesting that SWC measures implemented have significantly improved soil fertility and reduced soil loss. Therefore, it is necessary to scale up the SWC practices to other untreated areas of the watershed in the Blue Nile Basin of Ethiopia to achieve sustainable development.

## 1. Introduction

Due to the unwise use of land in the past, land degradation was a common challenge in Ethiopia [1–3]. This has led to the collapse of agriculture to feed the population [2], together with the depletion of the natural resources, which has made food insecurity and dependence on food aid over the last four decades [4–7]. Therefore, reversing this challenge is indispensable through restoration mechanisms implemented at the in‐country level based on agroecology, land types, and effectiveness [8, 9].

Moreover, over the last four decades, large‐scale food aid deliveries and many soil and water conservation (SWC) activities have been implemented in various parts of Ethiopia to maintain land productivity and overcome food security challenges through a watershed‐level approach, funded by external sources, with the Ethiopian government’s initiatives and responsibility to implement the projects. For instance, Food‐for‐Work: Managing Environmental Resources to Enable Transition to more sustainable livelihoods (MERET) (2003–2015), Productive Safety Net Programs (PSNP) (2005–present), community mobilization through free labor days (1998–present), and the National Sustainable Land Management Project (SLMP) (2008–present) [10–12].

Ethiopia’s food security is seriously threatened by land degradation, particularly soil erosion [13], as land degradation has been the main threat to soil quality in Ethiopia [14, 15].

To mitigate these problems across Ethiopia, the country’s government has implemented successive SWC practices over the past 40–50 years [16]. The Ministry of Agriculture (MoA) in Ethiopia has undertaken extensive SWC efforts on degraded land by constructing bunds and hillside terraces combined with afforestation, among others [17].

Farmers must be made aware of the measures that can be used to limit runoff, soil and nutrient losses, and the impact on crop output to implement SWC practices [18]. Even though intensive restoration work has been conducted across the Ethiopian highlands for decades, the scientific literature on the impacts of restoration on soil erosion, sediment export, vegetation improvement, and other ecosystem services has not been systematically reviewed [19].

Additionally, conservation and soil management interventions need to be planned with an understanding of the landscape position, which critically influences soil quality and soil loss across various land‐use types [20].

According to a previous study in the Blue Nile, including the Fetam watershed, soil erosion is severely affecting the area [21], and various SWC measures have been implemented. The loss of vegetation cover in this area due to grazing and deforestation has predisposed the farm fields to severe soil erosion and reduced soil fertility.

To address soil erosion and improve soil fertility, SLMPs under the MoA have been implemented to restore degraded land and replenish soil nutrients. However, the impacts of various SWC practices on reducing soil erosion and enhancing soil fertility have not been studied or documented in detail.

The annual soil erosion rate is estimated at 42 t/ha in the Ethiopian highlands [22, 23], which is well above the tolerable soil erosion rate [24]. The study watershed is one of the upper Blue Nile basins that contribute a large amount of sediment to Lake Tana, Ethiopia. As a result of soil loss, the soil fertility of croplands decreases, which, in turn, reduces crop yields. Farmers apply fertilizer to grow crops, but runoff also washes it away, reducing its benefit to soil fertility and maximizing crop yield.

The sustainable land management (SLM) program has been implemented to improve soil health and fertility interventions in the study area. However, the program needs to understand the effects of the interventions on soil loss and soil fertility in the Fetam watershed in northwest Ethiopia. This research shows the impact of the restoration and nonrestored areas in treated (Hunkan) and untreated (Kindikan) subwatersheds by quantifying biophysical environment such as soil erosion, sediment export, and soil fertility. This significantly helps in monitoring and evaluating the project implementation.

The specific objective of the study is to investigate the effects of SWC practices on selected soil physical and chemical properties, disaggregated by slope position, and to estimate soil loss resulting from restoration activities.

Section 1 presents the study’s introduction and background. Section 2 explains the materials and methods, and Section 3 presents the research results, followed by a discussion of the key findings in Section 4. Finally, Section 5 presents the study’s conclusions and recommendations.

## 2. Materials and Methodology

### 2.1. Description of the Study Area

**FIGURE 1:**
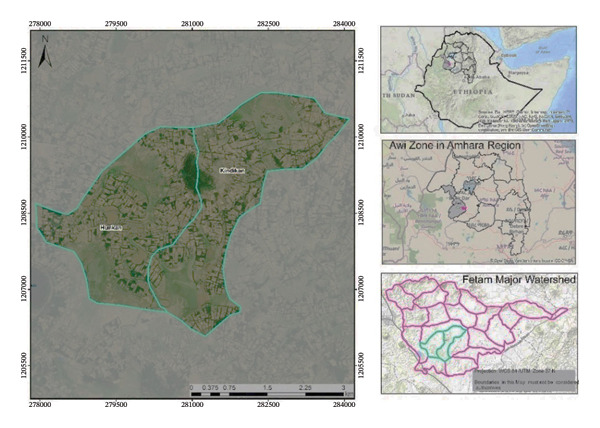
Location of the study area, showing treated (Hunkan) and untreated (Kindikan) subwatersheds.

The study was conducted in the Fetam watershed of Banja District, Amhara Regional State, northwest Ethiopia, and it is part of the Blue Nile Basin (Figure 1). Geographically, the watershed is located at 12°53′15″N and 37°59′42″E, with altitudes ranging from 2611 to 2889 m above sea level.

**FIGURE 2:**
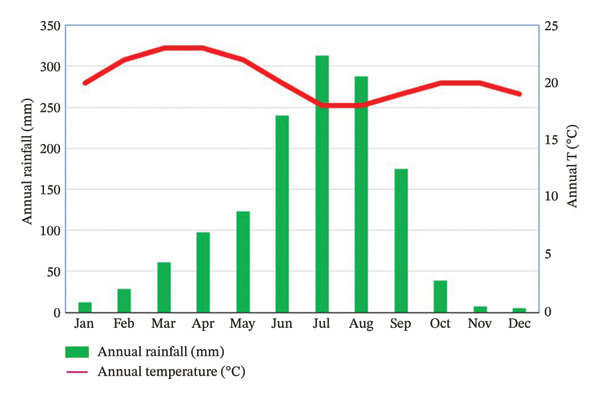
Mean monthly rainfall and temperature.

Based on data from the Ethiopian Meteorological Institute (EMI), the study watershed has three meteorological stations (Injibara, Tilili, and Sekela). Fetam watershed falls in the “Dega” agroecological zone (AEZ) with an average temperature ranging from 18°C to 24°C, and the annual rainfall amount of the Year 2022 exceeded 1500 mm (Figure 2), with maximum rainfall amount in June, July, and August.

The majority of the watershed shows an average slopes of 15%, indicating predominantly flat to gently sloping terrain. In comparison, areas with slopes greater than 30% occupy only a small proportion of the watershed. This indicates that the topography of watersheds is diverse, with hills, valleys, and plateaus, making the watershed’s landscape unique.

Farmers depend on a mixed crop–livestock production system in which livestock provide traction power, transportation, and manure for crop cultivation. Livestock sales provide finances to buy fertilizers to maintain soil fertility. In turn, the crop sector provides feed for animals through crop residues. In the homestead areas, vegetables, mainly Irish potatoes (*Solanum tuberosum*) and cabbage, are cultivated. Different types of tree species are also grown in the area, such as acacia (*Acacia abyssinica*), Bamboo (*Bambusa vulgaris*), Eucalyptus (*Eucalyptus globulus*), wanza (*Cordia africana*), isana (*Croton macrostachyus*), and yehabesha tid (*Juniperus procera*). Various wild animals and birds live in the forests around the subwatersheds, including apes, hyenas, foxes, and tigers.

### 2.2. Data Sources and Data Collection Method

The study explored the impacts of SWC measures on selected soil physical and chemical properties, disaggregated by landscape position, and estimated soil erosion using a model to compare the treated and untreated subwatersheds.

#### 2.2.1. Integrated Valuation of Ecosystem Services and Tradeoffs (InVEST) Model for Soil Erosion Estimation

The InVEST sediment delivery ratio (SDR) model is used to quantify and map soil loss in a landscape [25]. To simulate the model, the required parameters included land‐use/land‐cover data, Revised Universal Soil Loss Equation (RUSLE) factors, digital elevation model (DEM), maximum SDR, Borselli’s parameters, watershed and subwatershed boundaries, and the biophysical table. Each parameter was prepared according to the data requirements of the InVEST modeling [26]. All spatial inputs must use the same projected coordinate system (linear units in meters). The biophysical table contains the values of cover management factors (C) and conservation practice factors (P) associated with each land‐use/land‐cover code.

##### 2.2.1.1. Model Input Parameters

###### 2.2.1.1.1. Land Uses

This study used Level 2 surface reflectance (SR) products from the Landsat 5 TM and Sentinel 2 sensors available in Google Earth Engine (GEE). Three sets of digital satellite images from the Landsat 5 TM and Sentinel 2 sensors for 2009, 2016, and 2023 were used to examine LULC dynamics. The years of analysis were deliberately selected to align with the significant impact of SLM interventions and noninterventions. The majority of the image processing and analysis for the study was conducted using the GEE application programming interface (API). All datasets used in this study are freely available on GEE. Therefore, eight major LULC types were classified for the years 2009, 2016, and 2023. The selection of the initial year (2009) was based on the project’s start, and the remaining years (2016 and 2023) were used to monitor and evaluate biophysical factors after the project’s implementation. The overall accuracy of this study is consistent with that reported by Tadesse et al. [9], who found an overall accuracy of 80% or higher. Hence, the overall accuracy was within the acceptable range for further LULC change analysis.

To simulate soil loss in the watershed using the InVEST model, the RUSLE equation (Equation (1)) was used.

(1) SLi=Ri.Ki.LSi.Ci.Pi,

where SL_i_ is mean annual soil loss (t/ha/year), Ri is the rainfall erosivity (MJ Mm/ha/year), Ki is the soil erodibility (MJ mm/t/ha), LSi is the topographic factor (dimensionless) (length and gradient), Ci is cover (dimensionless) simulated utilizing Normalized Difference Vegetation Index (NDVI), and P is land management factors (dimensionless).

###### 2.2.1.1.2. Erosivity (R Factor)

The erosivity factor was calculated using Equation (2), as provided by Hurni [27], and derived from spatial regression analysis for Ethiopian highlands based on the available mean annual rainfall (P) in the study watershed. These data are available monthly and need to be updated annually to ensure that the InVEST model is suitable for each reference year (2009, 2016, and 2023).

(2) R=−8.120.56+∗P,

where *R* is the erosivity factor and *P* is the mean annual rainfall (mm/year).

###### 2.2.1.1.3. Erodibility (K Factor)

The erodibility (K factor) was computed for each soil type using Equation (3) adopted from Williams [28] and performed in ArcGIS 10.5 using a raster calculator. The Ethio‐GIS server (https://www.ethiogis-mapserver.org/) was used to get the soil types. The soil’s physical properties were determined in the laboratory.

(3) KRUSLE=fcs and×fcl−si×forgC×fhisand,

where fcsand refers to a factor that provides soil erodibility of low and high value for soils of high coarse‐sand and little sand contents, respectively; fcl‐si represents soil erodibility factors that are low value for soils rich in a high clay‐to‐silt ratio; forgC is a factor that decreases soil erodibility with organic carbon (OC) in high content; and fhisand is a factor that reduces soil erodibility with extremely high sand contents.

###### 2.2.1.1.4. Cover Factor (C Factor)

According to Durigon et al. [29], the corresponding average C value was determined for each LUC class for the reference years (2009, 2016, and 2023) based on NDVI values using Equations (4) and (5).

(4) C=−NDVI+12,

(5) NDVI=NIR−REDRED+NIR,

where C is the cover factor, NIR is the surface spectral reflectance in the near‐infrared band, and RED is the surface spectral reflectance in the red band.

The NDVI is a useful technique to distinguish vegetation and nonvegetation areas in the landscape. It was computed by processing Landsat images using the GEE API.

###### 2.2.1.1.5. Land Management Practices Factor (P)

This was determined based on conservation practice types. The various SWC practices were implemented, reviewed, and collected from different kinds of literature. Depending on conservation practices, with or without vegetation cover, the P factor ranges from 0 to 1 [27, 30]. The highest *p* values indicate good conservation practices, whereas the lowest indicate that the land requires them. Finally, the spatial and temporal changes in soil loss were estimated based on the initial year (2009), the intermediate year (2016), and the final year (2023) to show the impact of the SLM project.

#### 2.2.2. Assessment of Soil Erosion in the Untreated Subwatershed

Owing to deforestation, overgrazing, and repeated cultivation, combined with rugged terrain and inappropriate management practices, severe soil erosion problems have been triggered in the Fetam watershed. The untreated Kindikan subwatershed shows the severity of soil erosion, with gullies forming across much of the landscape. This soil loss has also led to reduced soil fertility and increased sedimentation in the lower catchment and river bodies. Deforestation, the removal of trees and other vegetation, has led to biodiversity loss and increased soil erosion in untreated watersheds.

#### 2.2.3. Assessment of the Implementation of SWC Practices in the Subwatershed

In recent years (2009–2022), SWC practices were implemented in the Hunkan subwatershed. As indicated in Table 1, the SWC interventions are predominantly physical measures, including gabion check dams, soil and stone bunds, hillside terraces, drainage ditches, water harvesting, and area closure. In certain cases, biological measures are combined with physical measures, including grass strips (e.g., elephant grass) and the planting of fruit or fodder trees within the conservation structures. Biological conservation measures strengthen and stabilize structural measures while also providing useful products, such as animal feed or household wood. These measures cover about 160 ha of the Fetam watershed (650 ha in total), thus indicating that SWC measures have so far covered only 25% of the Hunkan subwatershed (Table 1).

**TABLE 1:** Implemented biophysical SWC measures in the Fetam watershed.

SWC measures implemented	Area treated (ha)	Dominant landscape position
Hillside terrace	30	Upper landscape position
Contour (level) bund	11	Both landscape positions
Tree planting	20	Both landscape positions
Grass strips	15	Upper landscape position
Area closures	77	Both landscape positions
Check dam	5	Upper landscape position
Total treated area	160	Both landscape positions

Abbreviation: MoA, Ministry of Agriculture.

#### 2.2.4. Soil Sampling

The Fetam watershed is disaggregated into treated (Hunkan) and untreated (Kindikan) subwatersheds and divided into upper and lower catchments based on slope categories. Upper slopes are defined as landscapes with a slope gradient above 30%, while lower slopes are those with a slope gradient below 15%. The landscape between 15° and 30° slopes is considered a middle or transporting landscape, while the upper slope is considered eroding, and the lower slope is considered a deposition landscape. Based on this systematic disaggregation of the watersheds, eight composite soil samples were collected, one from each of the upper treated and upper untreated watersheds. Similarly, eight samples were collected from lower‐treated and lower‐untreated subwatersheds, giving a total of 32 topsoil (0–20 cm) samples. The samples were collected using an Edlam hand‐drilled auger using a systematic sampling method.

#### 2.2.5. Soil Laboratory Analysis

Soil samples were taken to the soil fertility laboratory of Amhara Water Works Design Supervision Enterprise, located in Bahir Dar, Ethiopia, to determine the soil’s physical (texture and bulk density [BD]) and chemical (pH, OC, total nitrogen [TN], available phosphorus [AP], exchangeable bases, and CEC properties. The samples were air‐dried, well‐mixed, and passed through a 2‐mm sieve [31]. Soil texture (sand, silt, and clay proportions) was determined using the Bouyoucos hydrometer [32]. BD was determined by drying the soil sample at 105°C in an oven for 24 h, using the Blake and KH [33] procedure. BD is a mass of soil divided by core volume, as shown in Equation (6).

(6) BD=M÷V,

where BD is bulk density (g/cm^3^), M is the mass of oven‐dry soil (g), and V is the volume of core (cm^3^).

Soil chemical properties (pH, OC %, CEC, exchangeable bases, and NPK) were tested. Soil pH (H_2_O) was determined potentiometrically in deionized water at a soil‐to‐water ratio of 1:2.5, following the procedure. The soil OC (%) [24] was determined using the Walkley and Black wet combustion method [35]. Using the percolation approach and the ammonium acetate (pH 7) method, the CEC was analyzed. AP (ppm) was analyzed using the sodium bicarbonate extraction solution method (pH 8.5) [36]. According to Bremner and Mulvaney [37], TN in soil samples was determined using the Kjeldahl method. Exchangeable cations (Ca^2+^, Mg^2+^, K^+^, and Na^+^) were determined using an atomic absorption spectrophotometer (AAS) and the ammonium acetate extraction method [38].

### 2.3. Statistical Analysis

In SPSS Version 26, Student’s *t*‐tests were performed to compare mean differences between treated and untreated subwatersheds. In addition, one‐way analysis of variance (ANOVA) was used to analyze the effect of slope positions on the physical and chemical properties of soil. Mean separation was performed using least significant difference (LSD) at the 95% confidence level. Subwatersheds (treated and untreated) and landscape positions (upper and lower) were independent variables. Hence, selected soil physical and chemical properties were treated as dependent variables in this research. The relationship between the physicochemical characteristics of each soil was examined using Pearson’s correlation.

## 3. Results

### 3.1. Soil Loss in the Treated and Untreated Watersheds

**FIGURE 3:**
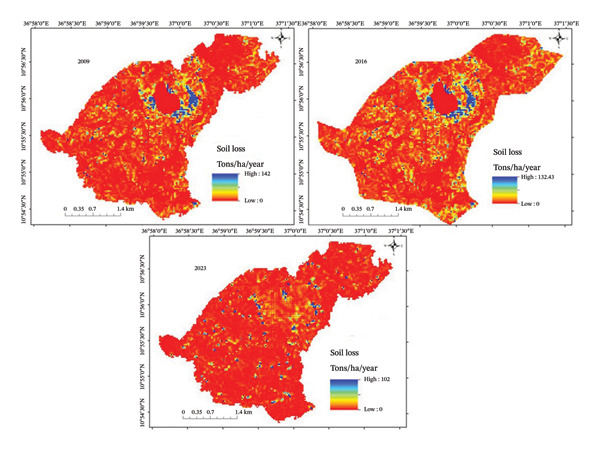
The trend of soil loss rates using the InVEST–SDR model t/ha/year.

The InVEST–SDR model result shows that the soil loss rate were significantly different between the untreated and treated subwatersheds (*p* > 0.05) (Figure 3). The mean soil loss in the treated and untreated microwatersheds was 14.43 ± 6.63 and 20.31 ± 10.28 t/ha, respectively, which indicated that the soil loss in the untreated watershed was increased by 5.88 t/ha as compared to the treated watershed. Similarly, the sediment export is lower in the treated watershed. This indicates that the SWC conservation structures implemented to restore degraded land contributed to reducing soil erosion rates.

A significant variation in soil loss across land‐use/land‐cover types, both treated and untreated, was observed (*p* > 0.05) (Table 2). Soil loss increased from 29.99 t/ha on the treated land to 43.42 t/ha on the untreated area of the farm land, a difference of 69%. In other words, soil loss in the treated farmland was lower than in the untreated farmland due to restoration interventions implemented over the last 15 years, including soil bunds, bench terraces, cutoff drains, and other biological and physical SWC practices. For each land‐use/land‐cover type, soil loss declined in the treated landscape compared to the untreated area. Moreover, soil loss on forest land is lower than that on farmland due to the vegetation’s protective capacity.

**TABLE 2:** The soil loss and sediment export (t/ha) in land use/land cover.

Land use	Treated area		Untreated area	
	Soil loss	Sediment export	Soil loss	Sediment export
Forest	3.76	1.43	4.89	2.1
Farmland	29.99	14.21	43.42	22.76
Grazing	14.53	9.35	19.35	8.54
Built‐up	10.23	8.43	14.21	9.9
Wetland	0.09	0.04	5.9	3.23
Bare land	34.65	20.32	39.65	25.35
Shrubland	14.54	8.32	22.88	12.61
Woodlot	7.69	3.45	12.21	3.54
Overall	14.43	8.19	20.31	11.00

The overall trend of soil loss also shows a decline in the intervention subwatershed in the last 15 years, from 17.54 in 2009 to 14.43 t/ha in 2023. The decline in soil loss was due to SWC interventions, while the trend of loss in the untreated land use increased from 17.34 t/ha in 2009 to 20.31 t/ha in 2023 due to the absence of interventions.

### 3.2. Effect of SWC Treatments on Soil Properties

#### 3.2.1. Effects of SWC Practices on Particle Distribution and BD

Notable differences were observed between treated and untreated subwatersheds in the soil particle distributions of silt and clay, but not in sand (*p* ≤ 0.05). The treated subwatershed (37 ± 1.04) recorded a higher mean silt value, while the untreated subwatershed (28.12 ± 1.04) recorded a lower mean silt value. As compared to the treated (23.12 ± 2.59) subwatershed, greater mean values of clay texture were found in the untreated (38.25 ± 2.59) subwatershed (Table 3).

**TABLE 3:** Mean comparison of soil physical properties for treated and untreated subwatersheds (*t*‐test).

Physical properties	Treated (Hunkan)	Untreated (Kindikan)	Mean	Sd. error	F‐value	*p* value
Sand (%)	39.87	33.62	36.75	2.25	11.29	0.17
Silt (%)	37.00	28.12	32.56	1.04	1.31	0.00
Clay (%)	23.12	38.25	30.68	2.59	23.31	0.03
BD (g/cm^3^)	1.05	1.21	1.13	0.01	0.08	0.00
Texture class	Loam	Clay loam	Clay loam			

The treated subwatershed is classified as loam under the USDA method of soil texture classification, while the untreated subwatershed is classified as clay loam. Hence, due to conservation structures, the treated subwatershed contained an equal proportion of sand, clay, and silt textures. Six different classifications of soil texture are present, including loam, sandy loam, sandy clay loam, heavy clay, clay loam, and clay (Figure 4). Those soil texture classifications are quite significant. Understanding particle distributions is crucial because soil texture is a major physical attribute that affects water infiltration, soil loss rate, and plant growth. They refer to the relative proportions of sand, silt, and clay particles in the soil.

FIGURE 4Distributions of soil texture (a), BD (b), soil pH (c), and OC (d) in the subwatersheds.

**Figure:**
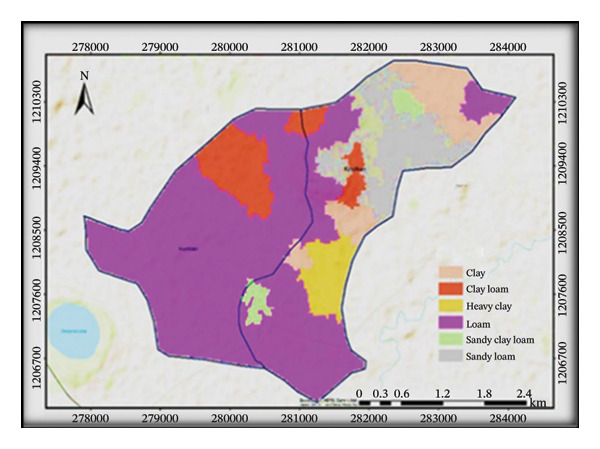
(a)

**Figure:**
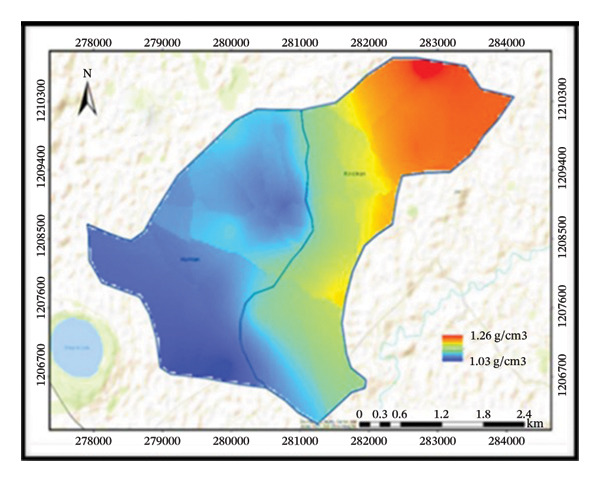
(b)

**Figure:**
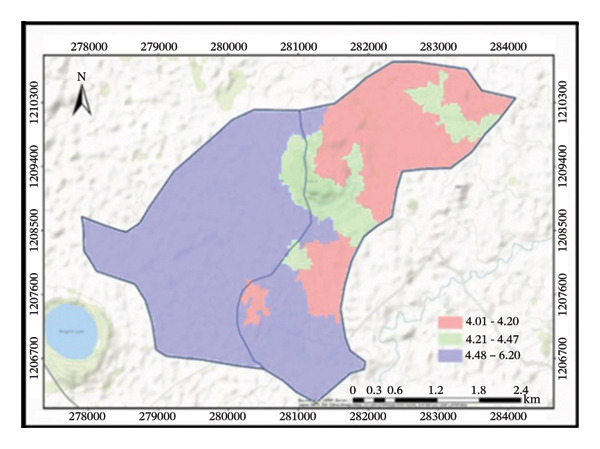
(c)

**Figure:**
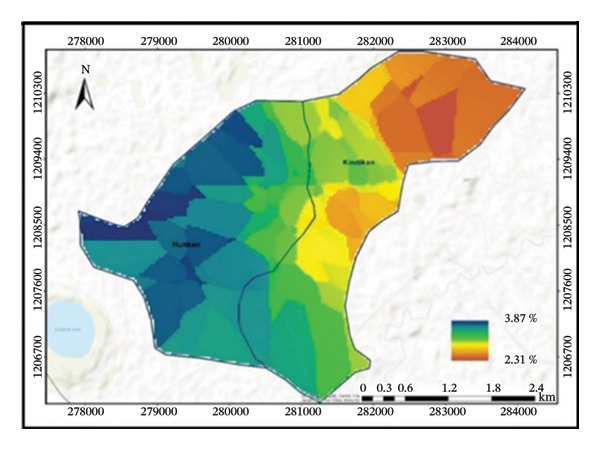
(d)

Between the treated and untreated subwatersheds, BD showed a highly significant difference at *p* ≤ 0.01. This indicates that untreated subwatersheds (1.21 ± 0.01) recorded higher mean BD values, whereas treated subwatersheds (1.05 ± 0.01) reported lower mean BD values (Table 3). On the other hand, SWC practices can reduce soil erosion, and the BD and soil textural distribution in the treated subwatersheds have improved.

#### 3.2.2. Effects of SWC Treatment on Soil pH, OC, TN, and AP

Soil pH, OC, TN, and AP showed statistically significant differences (*p* ≤ 0.05) between treated and untreated subwatersheds (Table 4). The findings for each parameter indicate that treated subwatersheds had higher mean values, whereas untreated subwatersheds had lower mean values (indicating poor soil fertility).

**TABLE 4:** Mean comparison of soil chemical properties for treated and untreated subwatersheds (*t*‐test).

Chemical properties	Treated (Hunkan)	Untreated (Kindikan)	Mean	Sd. error	F‐value	*p* value
pH (H_2_O)	5.89	4.19	5.04	0.15	8.81	0.00
OC (%)	3.69	2.35	3.02	0.23	25.57	0.04
TN (%)	0.30	0.18	0.24	0.01	21.70	0.01
AP (ppm)	22.78	7.69	15.23	3.2	32.7	0.20

Slope position influences soil chemical characteristics. Soil pH, OC, TN, and AP show statistically significant variations at *p* ≤ 0.05. For instance, soil pH showed higher mean values in the lower slope and lower mean values in the upper slope (Table 5). In addition, higher mean OC values were observed at lower‐slope positions within the subwatershed. When comparing treated and untreated subwatersheds within upper‐ and lower‐slope classes, TN shows statistically significant differences (*p* ≤ 0.05). In both treated and untreated subwatersheds, AP showed significant differences (*p* ≤ 0.05) between upper‐ and lower‐slope classes. AP has a higher mean value in the lower‐slope than in the upper‐slope classes (Table 5).

**TABLE 5:** Effect of SWC treatment and slope position on soil pH, OC, TN, and AP.

Subwatersheds	Slope position	pH (H_2_O)	OC (%)	TN (%)	AP (ppm)
Treated (Hunkan)	Upper	5.65	3.32	0.27	2.07
	Lower	6.19	3.98	0.32	38.88
	Mean	5.89	3.69	0.30	22.78
	SE	0.07	0.13	0.01	5.60
	F‐value	0.01	2.07	1.93	9.15
	*p* value	0.00	0.00	0.00	0.00

Untreated (Kindikan)	Upper	4.04	1.00	0.07	2.67
	Lower	4.34	3.70	0.28	12.71
	Mean	4.19	2.35	0.18	7.69
	SE	0.04	0.38	0.02	1.86
	F‐value	5.06	14.88	8.51	8.91
	*p* value	0.00	0.00	0.00	0.00

#### 3.2.3. Effects of SWC Treatments on CEC and Exch. Bases

There is no significant difference between CEC and exchangeable bases at *p* ≥ 0.05 (Table 6) between the treated and untreated subwatersheds studied. This might be because the two subwatersheds may have similar geological or parent material composition.

**TABLE 6:** Mean comparison of soil chemical properties for treated and untreated subwatersheds (*t*‐test).

Chemical properties	Treated (Hunkan)	Untreated (Kindikan)	Mean	Sd. error	F‐value	*p* value
CEC (cmol(+)/kg	32	31	31	0.56	3.74	0.21
Ca_2_^+^	22	21	20	0.14	0.04	0.51
Mg_2_^+^	8.0	7.0	7.5	0.04	1.25	0.77
Na^+^	0.09	0.08	0.09	0.01	1.15	0.12
K^+^	0.60	0.50	0.55	0.03	0.33	0.19

On the treated and untreated slopes, the subwatershed position class (upper and lower) shows significant variation at *p* ≤ 0.05. Hence, CEC mean values are higher in the lower‐slope position than in the upper‐slope position of the subwatershed (Table 7). It is used to hold and exchange positively charged ions, such as Ca^2+^, Mg^2+^, K^+^, and Na^+^, in the soil. Magnesium (Mg_2_^+^) shows significant differences at *p* ≤ 0.05 between treated and untreated slope positions. That means the Mg_2_^+^ mean value is higher in the lower‐slope position than in the upper‐slope position of the subwatershed (Table 7).

**TABLE 7:** Effect of SWC treatment and slope position on CEC and exch. bases.

Subwatersheds	Slope position	CEC	Ca^2+^	Mg^2+^	Na^+^	K^+^
Treated (Hunkan)	Upper	29.04	2.75	0.32	0.09	0.55
	Lower	34.62	2.99	0.80	0.10	0.64
	Mean	32.18	2.88	0.59	0.09	0.60
	SE	0.91	0.18	0.07	0.01	0.05
	F‐value	5.46	6.67	0.00	0.02	0.82
	*p* value	0.00	0.77	0.00	0.94	0.71

Untreated (Kindikan)	Upper	28.75	3.13	0.39	0.07	0.60
	Lower	32.80	2.23	0.75	0.08	0.41
	Mean	30.77	2.68	0.57	0.08	0.51
	SE	0.64	0.23	0.06	0.01	0.05
	F‐value	7.11	10.79	0.06	2.07	1.09
	*p* value	0.00	0.07	0.01	0.35	0.07

### 3.3. Effects of Slope Position on Soil Physical Properties

Clay soil texture revealed a significant difference (*p* ≤ 0.05) in the mean values: 23.5 ± 6.56 in the untreated upper zone and 53 ± 8.75 in the untreated lower zone of the watershed. Bar graphs with different letters differ significantly for soil particles in the upper and lower landscape positions within each subwatershed. However, silt soil textures have no significant differences (*p* ≥ 0.05) in the treated (upper and lower zones) watershed. Conversely, among untreated upper‐ and lower‐slope positions within a watershed, silt soil textures have shown significant differences (*p* ≤ 0.05). BD showed a significant difference between the treated upper‐slope (1.1 ± 0.05) and treated lower‐slope (1.01 ± 0.04) positions (*p* ≤ 0.05). In addition, it showed a significant difference between an untreated upper‐slope (1.26 ± 0.03) and lower‐slope (1.16 ± 0.05) class of watershed (*p* ≤ 0.05) (Table 8).

**TABLE 8:** Effect of SWC treatment and slope position on soil physical properties.

Microwatersheds	Slope position	Sand (%)	Silt (%)	Clay (%)	BD (g/cm^3^)	Texture class
Treated (Hunkan)	Upper	46.25	37.00	21.75	1.10	Clay loam
	Lower	33.50	37.00	24.50	1.01	Loam
	Mean	39.87	37	23.12	1.05	Loam
	SE	1.91	0.75	1.51	0.01	
	F‐value	0.26	0.07	7.65	0.37	
	*p* value	0.00	1.00	0.38	0.01	

Untreated (Kindikan)	Upper	47.25	25.00	23.50	1.16	Clay loam
	Lower	20.00	31.25	53.00	1.13	Clay
	Mean	33.62	28.12	38.25	1.21	Clay loam
	SE	4.01	1.15	4.24	0.01	
	F‐value	8.36	0.65	0.23	1.75	
	*p* value	0.00	0.03	0.00	0.00	

### 3.4. Correlation Among Selected Soil Physicochemical Properties

Person’s correlation coefficient revealed that TN significantly correlated with OC (*r* = 0.944), as shown in Table 9. This positive correlation and increase in TN values might be due to increased biomass production, increased litter quantity, and increased organic matter decomposition. Soil pH was positively correlated with OC (*r* = 0.376). AP and TN were highly correlated with OC (*r* = 0.553 and *r* = 0.944), respectively (Table 9).

**TABLE 9:** Person’s correlation matrix on soil physicochemical properties.

	**pH**	**Sand**	**Silt**	**Clay**	**Ca**_**2**_^**+**^	**Mg**_**2**_^**+**^	**Na** ^**+**^	**K** ^**+**^	**CEC**	**OC**	**TN**	**AP**	**BD**

pH	1												
Sand	0.399^∗^	1											
Silt	0.689^∗∗^	−0.103	1										
Clay	−0.591^∗∗^	−0.852^∗∗^	−0.070	1									
Ca_2_^+^	0.140	0.229	−0.074	−0.367^∗^	1								
Mg_2_^+^	−0.035	0.085	−0.117	−0.292	0.170	1							
Na^+^	0.266	−0.124	0.257	0.022	0.168	0.007	1						
K^+^	0.239	0.300	−0.086	−0.434^∗^	0.263	0.179	0.400^∗^	1					
CEC	0.038	−0.514^∗∗^	0.377^∗^	0.204	−0.055	0.219	0.226	0.140	1				
OC	0.376^∗^	−0.562^∗∗^	0.708^∗∗^	0.324	−0.178	−0.189	0.243	−0.161	0.517^∗∗^	1			
TN	0.443^∗^	−0.508^∗∗^	0.720^∗∗^	0.267	−0.185	−0.141	0.308	−0.039	0.496^∗∗^	0.944^∗∗^	1		
AP	0.241	−0.340	0.427^∗^	0.025	−0.031	0.533^∗∗^	0.290	0.079	0.641^∗∗^	0.553^∗∗^	0.562^∗∗^	1	
BD	−0.794^∗∗^	−0.617^∗∗^	−0.450^∗∗^	0.739^∗∗^	−0.203	−0.037	−0.128	−0.35^∗^	0.156	0.052	−0.053	0.048	1

^∗^Correlation is significant at the 0.05 level (two‐tailed).

^∗∗^Correlation is significant at the 0.01 level (two‐tailed).

In addition to those parameters, CEC and OC showed a significant correlation (*r* = 0.517). Also, BD with clay shows a highly significant correlation (*r* = 0.739) in the investigated subwatersheds. This might be because clay has highly closed pore spaces and a compacted texture.

## 4. Discussion

### 4.1. The Effect of SWC Practices on Soil Erosion

The study shows that the intervention, which applies different SWC practices in the treated watershed, has reduced soil loss and improved soil fertility. The study results show a similar pattern to that of Asnake and Elias [39], indicating that the stone‐faced soil bund is essential for soil moisture retention by reducing runoff velocity and conserving and storing water. In addition, Wolka et al. [40] found that soil bunds reduce soil erosion rates. This study clearly shows that SLM interventions improved ecosystem services, particularly by reducing soil erosion through the implementation of different SWC practices in the watershed.

SWC activities in upland areas can increase soil fertility and agricultural production in both upstream and downstream landscape positions [41]. Hence, conservation practices help preserve soil fertility, prevent nutrient loss, maintain soil structure, and reduce soil erosion in the study watershed.

In the study watershed, soil loss in the treated and untreated watersheds is 14.43 and 20.31 t/ha, respectively. This significant difference is due to the application of physical and biological SWC activities. A similar study by Abera et al. [42] showed that soil erosion decreased from about 12 to 7 t/ha/yr when conservation measures were implemented. This is supported by Schmidt et al. [43], who conducted a study in northwestern Ethiopia, showing that the implementation of terraces on mid‐ and high‐sloped areas would significantly decrease runoff and sediment yield.

### 4.2. Effect of SWC Practices on Soil Fertility

Overgrazing and soil erosion may be the cause of increased BD in untreated subwatersheds. However, SWC practices can alter soil physical and chemical characteristics and crop productivity [44]. The result concurs with the findings of Belayneh et al. [31], who studied the effects of SWC practices on soil physicochemical properties in the Gumara watershed of the upper Blue Nile Basin.

The study conducted by Jiru and Wegari [45] reported that BD was higher at untreated sites. Similarly, Bojago et al. [46] reported that the mean BD is higher in treated watersheds than in untreated watersheds. In the study watershed, higher mean BD values were recorded in the untreated subwatersheds, while the treated subwatersheds had lower values. This is due to the higher mean clay texture, which may be driving increased BD in untreated subwatersheds.

Soils with a high OM content tend to be more fertile and have better water‐holding capacity [47]. SWC measures improve soil pH, OM content, nutrient content, and CEC [48]. According to Bojago et al. [46], the mean soil pH was higher in conserved land and lower in unprotected land, suggesting that SWC practices have improved soil pH. A study conducted by Tolesa et al. [44] found significant differences in soil pH between watersheds treated with SWC measures, with treated watersheds having a more favorable soil pH than untreated ones.

The mean soil pH in the treated subwatershed is higher than in the untreated subwatershed. This might be due to SWC practices that positively affect soil pH by mitigating runoff and increasing organic matter. This finding was consistent with the research by Terefe et al. [49], who examined Geda watersheds in the Highlands of Ethiopia. Also, the study concurred with Ademe et al. [50], who found a lower mean pH in the absence of SWC, possibly due to lower base saturation and soil organic matter content, and a higher pH in its presence.

The study by Wolka et al. [40] reported that higher OC levels were recorded at the treated site in the Bokole watershed in the Dawuro region of southern Ethiopia. The mean OC values were higher in the treated subwatershed than in the untreated subwatershed, indicating that the treated subwatershed soil was improved due to the SWC intervention in the study watershed.

Organic matter shows significant differences between treated and untreated subwatersheds within slope classes. Higher mean OC values were observed in the treated and untreated lower‐slope positions of the subwatershed. This result might indicate that the upper‐slope fields are higher‐erosion areas, while the lower‐slope fields are settled areas with organic matter, plant, and animal residues. Most researchers report this finding. Thus, the result was similar to that of Elias [51], who noted that OC concentrations are higher on the lower slopes of watersheds. The result is also supported by Seifu et al. [20].

TN also shows significant differences across treated and untreated subwatersheds within slope classes. This result might be due to the removal of fertilizer from steep slopes by soil erosion, with deposition in lower‐slope zones. This finding is similar to that of Guadie et al. [14] and Elias [51], who reported that high TN was recorded in the lower slope than in the upper slope. Similar results were reported by Challa et al. [52] in the Zikre watershed in Ethiopia. Similarly, Aytenew [53] studied the Dawja watershed in northwest Ethiopia and reported that TN had a higher mean at lower elevations.

Implemented SWC activities have positively affected soil chemical properties. This visibly promotes greater exchange base and increases the availability of nutrients essential for plant growth [54]. According to Tolesa et al. [44], soil chemical properties were affected by SWC structures.

According to Dessie et al. [55], around 43.16% and 41.05% of households applied stone and soil bunds for soil fertility management in the northwest of Ethiopia. This study aligns with Yebo’s [56] findings, which revealed that smallholder farmers have adopted integrated soil fertility management strategies to improve soil fertility and crop output. This study indicates that integrated soil fertility management needs to be investigated further, along with a restoration of farm land management.

The study was conducted at the watershed level to improve SWC planning, but complex socioenvironmental factors also affect soil erosion and soil fertility, such as land use, population settlement, infrastructure, soil, slope, river proximity, curvature, and aspect [57]. Based on this issue, further research will be conducted to understand the overall restoration effect in the watershed.

## 5. Conclusion

Due to anthropogenic and natural factors, the study area’s landscape is severely affected by soil erosion and is at risk of soil fertility loss. To reverse these challenges, restoration activities were implemented in the study area. However, the restoration effect was not studied in detail. Therefore, this study investigated the effects of SWC practices on soil erosion and soil fertility status in the treated and untreated subwatersheds using field data collection, laboratory analysis, and modeling. The results show that the treated subwatershed (Hunkan) had a greater reduction in soil erosion and improved soil fertility than the nontreated subwatershed (Kindikan). Thus, improvements in soil physicochemical properties and reductions in soil erosion were positively influenced by SWC technology practices in the treated Hunkan subwatershed. In contrast, in the untreated subwatershed, soil fertility was lower than in the treated watershed. Therefore, SLM projects have made a significant contribution to improving soil fertility and reducing soil erosion.

The conserved Hunkan watershed is improving the ecosystem services due to interventions. At the same time, the untreated subwatershed is susceptible to severe degradation due to overgrazing, deforestation, and the absence of modern SWC practices, resulting in low fertility and increased soil erosion.

The InVEST–SDR model focuses only on analyzing sheet and rill erosion processes and does not quantify gully erosion. In many highland landscapes in Ethiopia, including the study watershed, gully erosion contributes considerably to the total soil loss and sediment yield. Therefore, holistic estimation of soil erosion, particularly gully erosion, is a future research area in the watershed.

Future research in the watershed, validation through field measurements, including setting up runoff plots, sediment, and hydrological monitoring at the gauging stations, remains necessary to improve accuracy and strengthen confidence in absolute values. Future research should focus on analyzing the temporal dynamics of ecosystem services, especially soil erosion and soil properties, by systematically comparing conditions before and after land management interventions to better quantify restoration impacts and sustainability outcomes.

Overall, the SWC practices initiated in the treated watershed of the study area should be continued and implemented in other nonconserved watersheds in Ethiopia to achieve SLM. Therefore, extension programs on SLM and awareness‐raising for communities and farmers about the importance of integrated watershed management need to be continued. At the same time, assessing the biophysical and socioeconomic impacts of these restoration interventions should be a mandatory component of all restoration projects implemented in Ethiopia.

Future research should focus on forecasting soil erosion across various land management scenarios. For improved conservation planning, the most sensitive characteristics that contribute to soil erosion (management factors) should be evaluated in various management situations. Furthermore, future research should examine the influence of soil loss on crop productivity and farmers’ perceptions of soil erosion in relation to restoration activities across farm management systems.

## Funding

No funding was received for this manuscript.

## Conflicts of Interest

The authors declare no conflicts of interest.

## Data Availability

The data that support the findings of this study are available from the corresponding author upon reasonable request.
